# Development and validation of a maternal anxiety for neonatal jaundice scale in China

**DOI:** 10.1186/s12888-022-04161-1

**Published:** 2022-08-04

**Authors:** Qin Yan, Dandan Li, Xiaoxv Yin, Nan Jiang, Na Sun, Qing Luo, Xin Pang, Lichun Fan, Yanhong Gong

**Affiliations:** 1 Department of Social Medicine and Health Management, School of Public Health, Tongji Medical College, Huazhong University of Science and Technology, No.13 Hangkong Road, 430030 Wuhan, P. R. China; 2 Department of Child Heath Care, Hainan Women and Children’s Medical Center, NO.15 South of Longkun Road, Haikou, P.R. China; 3 Department of Pediatrics, Hainan Women and Children’s Medical Center, NO.15 South of Longkun Road, Haikou, China

**Keywords:** Neonatal jaundice, Maternal anxiety, Scale validation, Delivery of health care

## Abstract

**Background:**

Maternal anxiety induced by neonatal jaundice has adverse effects on maternal and infant health, but there was no specific tool to identify the anxiety level of mothers. This study aims to develop a Maternal Anxiety for Neonatal Jaundice Scale (MANJS) and to validate it in the target population.

**Methods:**

An initial 11-items MANJS was developed through literature review, expert panel consultation, and a pilot-test. Subsequently, mothers of neonates with jaundice were recruited from the Maternal and Child Health Hospital of Hainan Province, China, from June to December 2018, for a formal questionnaire survey. Based on the data collected, the scale was validated for construct validity, convergent validity, discriminant validity, content validity, and internal consistency reliability after the items screening.

**Results:**

The reliability and validity of MANJS were validated in 1127 mothers of jaundiced neonates. After the item with cross-loadings was removed using exploratory factor analysis, MANJS consisted of two dimensions and 10 items, with a cumulative variance contribution of 74.36% and factor loadings above 0.6 for all items. The confirmatory factor analysis identified three items with cross-factor loading or error correlation and then they were removed orderly. The further confirmatory factor analysis showed a good construct validity for the 7-item MANJS, with standardized root mean square residual (SRMR) = 0.029, root mean square error of approximation (RMSEA) = 0.068, comparative fit index (CFI) = 0.961, Tucker-Lewis index (TLI) = 0.937, incremental fit index (IFI) = 0.961, normed fit index (NFI) = 0.954, goodness of fit index (GFI) = 0.998, adjusted goodness of fit index (AGFI) = 0.996, respectively. The average variance extracted values (AVE) of the two factors were 0.80 and 0.72, and the combined reliability (CR) were 0.94 and 0.88, respectively. Cronbach’s alpha was 0.90 for the MANJS, and split-half reliability was 0.72.

**Conclusions:**

MANJS was demonstrated to have satisfactory reliability and validity in evaluating maternal anxiety caused by neonatal jaundice among Chinese postpartum women.

**Supplementary Information:**

The online version contains supplementary material available at 10.1186/s12888-022-04161-1.

## Background

Neonatal jaundice is a common and frequent neonatal disease, most of which appears in the first week after birth. It can be seen in about 60% of full-term infants and over 80% of premature infants in the world [1]. Most cases of neonatal jaundice are physiological and do not require special treatment commonly as it can subside on its own [2]. However, in some children, jaundice may develop into acute bilirubin encephalopathy or nuclear jaundice, which is the primary cause of newborn re-hospitalization [3]. Although this condition is rare, it still needs to be monitored. Otherwise, the life and health of infants will be seriously endangered, and the surviving infant may have long-term neurodevelopmental sequelae [1, 3].

Newborn jaundice can also adversely affect mothers, especially on psychological health. The mental problem in postpartum women has received international attention for a long time, but compared to postnatal depression, there is still a need for more comprehensive research on postnatal anxiety as a separate event. A Meta analysis conducted in 2016 estimated that about 8.5% postpartum women overall were affected by one or more anxiety disorders, with a maximum of 39% [4], and more women were in a subclinical state of anxiety [5]. Anxiety is a complex psychological state. In 1966, Spielberger proposed the division of anxiety into trait anxiety and state anxiety [6]. Trait anxiety is a relatively stable personality trait with individual differences. Individuals with high levels of trait anxiety are more likely to perceive the threat posed by situational stress and therefore more inclined to experience higher levels of state anxiety [6, 7]. State anxiety is a temporary psychological or physical symptom that occurs in the face of a specific situation or stressful event [7, 8]. The degree and duration of maternal state anxiety is not only related to personal trait anxiety, but also depends on the role of some stressful events to some extent [9]. Neonatal health is usually an important event that causes maternal postpartum state anxiety [10], such as preterm birth, the infant admitted to the neonatal intensive care unit (NICU), and the appearance of neonatal jaundice in this study. Phillips et al. also find that 65% content of maternal worry was related to infant health, safety and wellbeing [11]. Due to the short mean length of hospital stay after delivery in most countries [12], a large number of neonatal jaundice symptoms appear or aggravate after discharge from hospital. However, many postpartum women have insufficient cognition of jaundice [13] and lack confidence in the knowledge and ability of nursing care after discharge [14], so they may become more worried about the condition of infants and be prone to common anxiety on neonatal jaundice [5].

The negative emotions of mothers may prevent them from adopting a correct attitude towards the problem of neonatal jaundice. It will also hinder the establishment of the mother-infant relationship, which will have a long-term impact on infants’ cognition, emotion, and physical development [15, 16]. The huge psychological pressure and burden of mothers is not conducive to postpartum recovery, may increase the risk of postpartum depression, and is also related to family harmony [17]. Meanwhile, temporary state anxiety in the early postpartum period is a key predictor of higher levels of maternal anxiety in long term [18]. In view of the above, the emotional impact of neonatal jaundice, which is highly prevalent in the early postnatal period, on mothers deserves attention. The scientific measurement of anxiety is undoubtedly an essential prerequisite to timely screen for the associated maternal state anxiety associated with neonatal jaundice, so as to provide targeted psychological interventions accordingly. Previous studies mainly focused on the mental intervention measures for the mothers of jaundiced neonates, and the measurement tools were mostly general scales, such as State-Trait Anxiety Inventory (STAI), Self-Rating Anxiety Scale (SAS), Hamilton Anxiety Scale (HARS), and Baker Anxiety Scale (BAI), etc [19–23]. There was no specific scale to measure maternal anxiety associated with neonatal jaundice. Therefore, this study aims to develop a Maternal Anxiety for Neonatal Jaundice (MANJS) to realize the targeted evaluation of the psychological status of postpartum mothers, so as to improve the scientificalness and effectiveness of psychological interventions.

## Methods

### Scale development

The development of MANJS was divided into three steps. In step 1, forming an item pool. The keywords “neonate”, “neonatal”, “health”, “jaundice”, “mother”, “maternal”, “parents”, “worry”, “anxiety”, “psychology” were used to search for Web of Science, PubMed, Google Scholar, China National Knowledge Infrastructure (CNKI), Wanfang Data. And we conducted comprehensive reviews of the literature about parent’s emotions and behaviors caused by neonatal health. The existing mature anxiety scales, such as the Hospital Anxiety and Depression Scale (HADS), the Self-Rating Anxiety Scale (SAS), and the State-Trait Anxiety Inventory (STAI) [23–25], were also referred to. In step 2, we organized a six-member panel of experts with relevant professional backgrounds, including two chief physicians of maternal and child health departments and one chief pediatrician with more than 10 years of clinical experience, one professor of psychology with expertise in maternal mental health, one professor of epidemiology and one professor of social medicine with extensive academic experience in designing similar measurement instruments. The experts discussed the importance, comprehensiveness, feasibility of these items according to their professional knowledge and practical experience, and finally extracted two factors conceptually associated with maternal anxiety for neonatal jaundice: (1) Physical and mental reaction; (2) Behavioral manifestation. Based on these two dimensions, 11 items were selected to form the preliminary MANJS. Third, the questionnaire was pilot-tested in 30 mothers of jaundiced neonates to ensure that the questions were clear and understandable to all participants, and the acceptability of the survey and the time required to complete it were also reviewed. These 30 mothers were recruited from the Maternal and Child Health Hospital of Hainan Province using the convenience sampling method.

### Scale validation

The validation study for the 11-item MANJS was conducted from June to December 2018, in the Maternal and Child Health Hospital of Hainan Province and the mothers of jaundiced neonates during postpartum hospitalization were recruited. The inclusion criteria of subjects were as follows: (1) the newborn was diagnosed by clinicians to have jaundice symptoms, (2) self-reported no history of psychiatric illness or substance use, (3) owned basic cognitive ability and understanding as judged by a trained nurse, (4) informed consent and willingly participated in this research. After the onset of neonatal jaundice, trained nurses explained the purpose and significance of this study to the mothers and obtained their cooperation. And questionnaires were filled in by the respondents themselves or explained to them by nurses and filled in on their behalf. The content of the questionnaire was divided into two parts: (1) basic demographic information, including the maternal age, educational level, residence, family economic status, parity, feeding pattern, and newborn gender, age, gestational age, type of delivery, etc. (2) the preliminary MANJS. Items of the MANJS were evaluated using the responses “never happened”, “occasionally”, “often” and “almost always” which were coded with values from 1 to 4. The total scale score was the sum of each item’s score. The higher the score, the more anxious the mother was about neonatal jaundice.

### Screening of scale items

#### Correlation analysis

The Pearson correlation coefficients between each item score and total MANJS score was calculated. And the higher the correlation, the higher the homogeneity between the item and the scale. If no statistical correlation was found between item score and total scale score (*P* > 0.05), the item was considered for screening out.

#### Discrimination coefficient

The scores of all the respondents to the 11-item MANJS in this study were sorted in order of high and low, with the top 25% respondents representing the high group and the bottom 25% representing the low group. An item’s discrimination coefficient was determined by the difference between the average scores of the item in these two groups. The greater the difference, the higher the discrimination power of the item. The item whose discrimination coefficient < 0.5 was removed from the scale.

#### Exploratory factor analysis

Exploratory factor analysis (EFA) was used to investigate the factor structure of the scale. Prior to EFA, Kaiser-Meyer-Olkin (KMO) > 0.5 or Bartlett’s spherical test *P* < 0.05 were used to determine the suitability of the data for factor analysis. Factor extraction was performed by principal component analysis and Varimax factor rotation. According to Kaiser criterion and Catell’s Scree plot, factors with eigenvalue greater than 1 were retained. Item removal criteria were: (1) the factor loading was less than 0.4 on each of factors, (2) items with substantial cross-loadings on two or more factors.

### Validity

#### Construct validity

Based on the factor structure extracted from the exploratory factor analysis, a confirmatory factor analysis (CFA) was conducted to explore the fitting between the factor structure of MANJS and actual data. The following assumptions were made about the CFA model: (1) no cross-factors loading, (2) errors were independent of each other. Due to the ordered categorical responses of the scale, weighted least square means and variance adjusted estimation (WLSMV) [26, 27] was chosen as the parameter estimation method to confirm the factor structure. We reported several goodness-of-fit indicators to assess overall model fit: χ^2^, df, χ^2^/df, standardized root mean square residual (SRMR), root mean square error of approximation (RMSEA), goodness of fit index (GFI), adjusted goodness of fit index (AGFI), Tucker-Lewis index (TLI), incremental fit index (IFI), normed fit index (NFI), and comparative fit index (CFI). According to the recommended cut-off value [28–31], χ^2^/df < 3, SRMR < 0.05, RMSEA < 0.08, GFI, AGFI, TLI, IFI, NFI, CFI > 0.95, were considered good fit values. And χ^2^/df < 5, SRMR < 0.8, RMSEA < 0.10, GFI, AGFI, TLI, IFI, NFI, CFI > 0.90 were considered acceptable fit indices. In addition, we examined the modification index and factor loadings, on the basis of the assumptions of CFA model, items with large error correlations and cross-factor loadings were removed, and items with factor loadings < 0.7 were also deleted. Removed one item at a time and performed CFA again until the fit indices reached expected levels.

#### Convergent validity and discriminant validity

The average variance extracted values **(**AVE**)**, combined reliability (CR), and correlation coefficients among factor scores were calculated to test the convergent validity and discriminant validity. AVE ≥ 0.5, CR > 0.7 and CR > AVE were demonstrative of an acceptable convergent validity [32, 33]. Discriminant validity was considered satisfactory if the correlation between factor scores was significant and the correlation coefficient was less than the square root of the corresponding AVE [33].

#### Content validity

It was assessed by Pearson correlation between the MANJS score and the sum scores for items within each factor.

### Reliability

#### Cronbach’s Alpha

This statistic was calculated to measure the internal consistency of the MANJS. Cronbach’s Alpha > 0.7 represented an acceptable reliability.

#### Split-half reliability

It was estimated by Pearson correlation between the scores of the odd and even items of the MANJS.

### Statistical analysis

All analyses were performed using the package Lavaan, semTool in R system (version 4.0.3) and IBM SPSS 25.0 (IBM Corp, Armonk, NY). For the descriptive analysis of general data, the enumeration data were expressed as frequency and constituent ratio (%), while the quantitative data were expressed as mean ± standard deviation (‾X ± S) or median and interquartile range. All the differences were tested using two-tailed tests and the significance level was set as 0.05.

## Results

During the study, a total of 1406 questionnaires were distributed and 1161 mothers of jaundiced neonates participated in the survey, with a response rate of 82.57%. 34 questionnaires were deleted due to the lack of data. Finally, we analyzed 1127 qualified questionnaires.

### Demographic characteristics

1127 mothers had an average age of 29.31 ± 4.64 years, and 45.52% of them were primiparous women, 74.53% were breastfed (Table 1). The proportion of male and female newborns was balanced, with an average age of 3.31 ± 2.39 days, and 64.06% of newborn were born in natural delivery (Table S1 in Additional file 1).

**Table 1:** Demographic characteristics of respondents (*N* = 1127)

Variables	n(%)/‾X ± S
**Age, year**	29.31 ± 4.64
≤ 20	22(1.95)
21 ~ 30	703(62.38)
31 ~ 40	384(34.07)
≥ 41	18(1.60)
**Educational level**	
Junior high school or lower	318(28.22)
Senior high school or technical secondary school	247(21.92)
Undergraduate or Junior College	535(47.47)
Postgraduate or higher	27(2.39)
**Residence**	
Urban	744(66.02)
Rural	383(33.98)
**Economic status**	
Very good	30(2.67)
Good	82(7.27)
General	974(86.42)
Poor	35(3.11)
**Parity**	
The first child	513(45.52)
The second child	521(46.23)
The third child or more	93(8.25)
**Feeding patterns**	
Breastfeeding	840(74.53)
Non-breastfeeding	28(2.49)
Mixed feeding	259(22.98)

### Screening of scale items

The correlation coefficients between the scores of 11 items and the MANJS score ranged from 0.65 to 0.83, all of which were significantly correlated (*P* < 0.001). And the discrimination coefficient of each item was greater than 0.5, indicating strong discrimination power. Therefore, all items were retained.

The Kaiser-Meyer-Olkin (KMO) value (0.89) and Bartlett’s spherical test (${x}^{2}$= 9859.405, *P* < 0.001) indicated that EFA could be performed on the scale data. Then principal component analysis and scree plot determined two factors, which explained 71.56% of the variance. After Varimax factor rotation, it was found that the factor loadings of item c2 (“Even if the doctor thought the child’s jaundice symptom was not serious, I could hardly relax”) were greater than 0.4 on the two factors, showing substantial cross-loading. As a consequence, this item was removed from the initial MANJS. The rotated factor loading matrix of 11 items is shown in Table S2 in Additional file 1.

The KMO value for the remaining 10 items was 0.87 and the Bartlett’s spherical test reported *P* < 0.001, so we performed EFA again. Table 2 presents the rotated factor loading matrix of 10-item scale. Factor 1 (Physical and mental reaction) contained 6 items of c3, c7, c8, c9, c10, and c11. Factor 2 (Behavioral manifestation) contained 4 items of c1, c4, c5 and c6. The variances of each factor explained were 54.87% and 19.53% respectively, and the cumulative percentage was 74.39%. These 10 items showed salient loadings on specific factors without substantial cross-loadings on the other factors.

**Table 2:** The rotated factor loading matrix of 10-item MANJS

Items	Factor loadings	
	F1	F2
**Factor 1: Physical and mental reaction**		
c3 I loss sleep at night or have nightmares because I am worried about my child’s jaundice.	**0.67**	0.26
c7 I feel easily irritated until my child’s jaundice subsides.	**0.83**	0.25
c8 I feel nervous until my child’s jaundice subsides.	**0.87**	0.25
c9 I feel restless until my child’s jaundice subsides.	**0.92**	0.18
c10 I have no appetite until my child’s jaundice subsides.	**0.91**	0.14
c11 I can’t concentrate on things because I am worried about my jaundiced child.	**0.88**	0.14
**Factor 2: Behavioral manifestation**		
c1 I am afraid that jaundice will threaten my child’s health.	0.28	**0.67**
c4 I go online for information about neonatal jaundice or keep consulting doctors and friends.	0.14	**0.86**
c5 I observe the child’s every move and repeatedly confirm whether the behavior was related to jaundice.	0.15	**0.89**
c6 I keep an eye on the child’s jaundice level and double check that it is within the normal range.	0.19	**0.84**
Eigenvalue	5.49	1.95
Variance explained (%)	54.87	19.53
Cumulative percentage (%)	54.87	74.39

### Validity

#### Construct validity

**Fig. 1:**
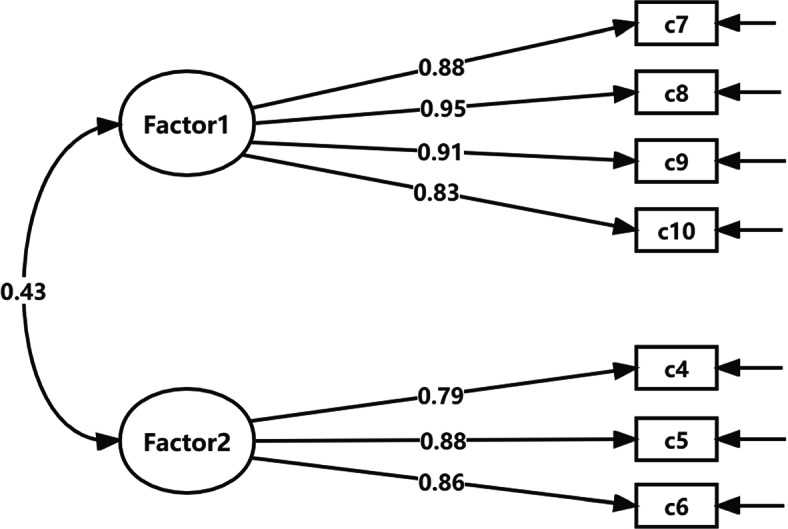
7-item confirmatory factor analysis path diagram

A CFA model was constructed based on the factor structure generated by EFA. Stepwise adjustments were made to the model according to the factor loadings and the modification index (Table 3). Firstly, the model fit of 10-item CFA was not yet satisfactory. c1 (“I am afraid that jaundice will threaten my child’s health”) had cross-factor loadings that violated the basic assumptions of CFA, and given that it was conceptually inconsistent with Factor 2 (Behavioral manifestation) which it belonged to, so c1 was removed. CFA was performed again using remaining items and fit indices still did not all meet the specified criteria. c3 (“I loss sleep at night or have nightmares because I am worried about my child’s jaundice”) was deleted due to a factor loading below 0.7. Furthermore, the modification index indicated a strong error correlation between c11 (“I can’t concentrate on things because I am worried about my jaundiced child”) and c10 (“I have no appetite until my child’s jaundice subsides”), and then c11, which had a low factor loading, was removed. Ultimately, the 7-item CFA model fitted best, with all fit indices achieving a desirable or acceptable level except for χ^2^/df (6.272), with factor loadings above or close to 0.8 for all items. The path diagram of 7-item CFA model is shown in Fig. 1. We also analyzed the correlations between items and the correlation matrix is available in Table S3 in Additional file 1.

**Table 3:** Fit indices for different CFA models

Models	χ^2^	df	χ^2^/df	SRMR	RMSEA	GFI	AGFI	TLI	IFI	NFI	CFI
10-item model	327.869	34	9.643	0.050	0.088	0.993	0.989	0.841	0.880	0.908	0.880
9-item model ^a^	201.946	26	7.627	0.039	0.078	0.996	0.994	0.887	0.919	0.908	0.918
8-item model ^b^	156.688	19	8.247	0.039	0.080	0.997	0.994	0.897	0.930	0.922	0.930
7-item model ^c^	81.534	13	6.272	0.029	0.068	0.998	0.996	0.937	0.961	0.954	0.961

^a^ Item c1 (“I am afraid that jaundice will threaten my child’s health”) was deleted

^b^ Item c3 (“I loss sleep at night or have nightmares because I am worried about my child’s jaundice”) was deleted

^c^ Item c11 (“I can’t concentrate on things because I am worried about my jaundiced child”) was deleted

### Convergent validity and discriminant validity

The AVE of the two factors were 0.80 and 0.72, and the CR were 0.94 and 0.88 respectively, the CR values in all factors were greater than AVE (Table 4). In addition, there was a significant correlation between the scores of two factors (*P* < 0.01), and the correlation coefficient was 0.43, which was less than the square root of the corresponding AVE (Table 5).

**Table 4:** Content validity and convergent validity of MANJS

Factors	Total Score		AVE	CR
	**r**	**P-value**		
Factor 1	0.89	< 0.0001	0.80	0.94
Factor 2	0.79	< 0.0001	0.72	0.88

r is Pearson correlation coefficient between the score of two factors and the total MANJS score

**Table 5:** Discriminant validity of MANJS

Factors	Factor1	Factor2
Factor1	0.80	
Factor2	0.43**	0.72
Square roots of AVE	0.89	0.85

Correlation coefficients were derived from CFA; **P < 0.01; Diagonal value was the AVE of each factor

#### Content validity

The correlation coefficients between the factor scores and the MANJS score were 0.89 and 0.79, respectively, indicating statistically significant correlations (*P* < 0.01) (Table 4).

### Reliability

Cronbach’s alpha was 0.90 for MANJS, and 0.94 and 0.86 for the two factors, respectively. Split-half reliability was 0.72.

### The MANJS score

The formal MANJS consists of 7 items with total scores ranging from 7 to 28 points. And the average score was 14.26 ± 4.77 among 1127 mothers, with a median score of 14.00 (10.00–17.00).

## Discussion

To compensate for the lack of relevant research, this study developed the first MANJS, focusing on a specific population of mothers of jaundiced neonates. The MANJS contains 7 items and a simple two-factor structure of “Physical and mental reaction” and “Behavioral manifestation”. Multi-step items screening, reliability and validity test verified that MANJS is a reliable and accurate measurement tool.

The preliminary MANJS consisted 11 items, and the discrimination coefficients were all greater than 0.5, demonstrating that it has sufficient discrimination power to distinguish different maternal anxiety levels for neonatal jaundice. EFA revealed the cross-loadings of c2 and deleted it. Finally, EFA extracted a factor structure of 2 factors and 10 items, with a cumulative variance contribution of 74.39%, which means that 2 factors can represent most of the information in the original scale.

The initial 10-item CFA model-to-data fit was inadequate. After sequentially removing item c1, c3 and c11 that had cross-factor loadings or correlated errors, most of the fit indices in 7-item CFA model met acceptable criteria except for χ^2^/df. As χ^2^/df tends to become larger with the increase of sample size [28], the large of this indicator may be due to the large sample size in this study. Meanwhile, considering that each item has a high factor loading on the factor to which it belongs. Overall, it can be concluded that the factor structure model of the 7-item scale fitted the actual data well and the scale has a good structural validity. CR values of two factors were about 0.9, indicating that the scale has good stability in reflecting the measurement results. And the AVE value of each factor was more than 0.5, which also supports the ideal convergence validity. Meanwhile, the correlation coefficient of the two dimensions was less than 0.5 and lower than the corresponding square root of AVE, hence there was a certain correlation and a certain degree of differentiation between the two dimensions, indicating that the MANJS has satisfactory discriminant validity [34, 35]. Furthermore, there were strong correlations between 2 factor scores and the total scale score, indicating that the two dimensions and MANJS reflected essentially the same content, which validates the content validity of the scale to some extent.

Reliability is used to reflect the stability and consistency of the scale’s measurement results. Relevant studies point out that if the length of the scale is too short, the value of Cronbach’s alpha will be reduced [36]. Although there are only 7 items in MANJS, Cronbach’s Alpha of the entire scale was far beyond the acceptable value, as were the Alpha values for the two factors, and the split-half reliability was above 0.7, reflecting the good internal consistency of MANJS.

The 2 factors of 7-item scale reflect the psychophysiological reactions and externalized behaviors of mothers for neonatal jaundice, respectively, which is largely consistent with the theoretical dimensions initially set out. The emotional anxiety and somatic anxiety are both part of the former dimension. Due to traditional cultural and social attitudes, Chinese express their emotions in a more euphemistic manner, while public stigma and self-stigma of mental illness are prevalent [37], thus individual emotions are often expressed through somatic forms. The latter dimension reflects mother’s behavior in seeking security and dependence in the face of neonatal jaundice.

As a specific tool for the assessment of maternal anxiety for neonatal jaundice, MANJS has prominent advantages. On the one hand, all the validation indicators showed that MANJS has good reliability and validity, which means that it can accurately evaluate the anxiety level of the mothers. On the other hand, during the scale preparation period, we took full account of the physical condition of the postpartum women. The final scale is relatively short, so it is highly practical. Accordingly, the MANJS is qualified to be a routine screening tool for the psychological condition of postpartum mothers in clinical care practice. Besides, we emphasize the measurement of maternal anxiety under specific circumstances (neonatal jaundice), which is not only conducive to identify the source of anxiety, but also conducive to the provision of personalized postpartum care, and it is of great significance for the maintenance of postpartum mental health.

Several limitations in this study should be noted. Firstly, we were unable to examine the criterion-related validity of the MANJS due to the lack of a “gold standard” to assess maternal anxiety for neonatal jaundice. Meanwhile, the interval of repeated measurement is generally 2 weeks for test-retest reliability. Considering the short duration of jaundice in most neonates, we did not obtain test-retest reliability of the scale. Secondly, in the process of CFA, 3 items were removed, mainly due to the existence of correlated errors and cross-factor loadings, which result in certain discrepancies between the final 7-item MANJS and the original scale. In the future, further refinement of MANJS within the conceptual and theoretical framework of anxiety may be required. Thirdly, the χ^2^/df of CFA model failed to meet statistical requirement. To verify the issue that χ^2^/df is susceptible to sample size, we randomly selected 500 and 300 samples from the total sample of 1127 for CFA, and found that the χ^2^/df of 7-item model dropped to 3.467 and 2.915 respectively, while other indices remained relatively stable (Table S4 in Additional file 1). Therefore, it can be demonstrated that sample size is the main possible reason for the high χ^2^/df in this study. Fourthly, pre-survey and formal questionnaire survey were only carried out in the Maternal and Child Health Hospital of Hainan province, so the samples may lack adequate representativeness, which limited the generalizability of the MANJS. Therefore, it is necessary to validate the scale in a larger population before it was widely used in care practice.

## Conclusions

The MANJS developed in this study has good psychometric properties in Chinese postpartum women who had a jaundiced neonate. The scale contains two dimensions of “physical and mental reaction” and “behavioral manifestation”, and it can be used for timely screening for the maternal anxiety caused by neonatal jaundice, thus helping to provide personalized and targeted intervention measures to them. This study fills the gap of scale research in this field, but it still needs to be validated in a larger, especially cross-cultural target populations.

## Supplementary Information

**Additional file 1.**

## Data Availability

The datasets generated and/or analyzed during the current study are not publicly available due to limitations of ethical approval involving the patient data and anonymity but are available from the corresponding author on reasonable request.

## Abbreviations

MANJS: Maternal Anxiety for Neonatal Jaundice Scale
NICU: Neonatal intensive care unit
STAI: State-Trait Anxiety Inventory
HADS: Hospital Anxiety and Depression Scale
SAS: Self-Rating Anxiety Scale
EFA: Exploratory factor analysis
CFA: Confirmatory factor analysis
GFI: Goodness of fit index
AGFI: Adjusted goodness of fit index
SRMR: Standardized root mean square residual
RMSEA: Root mean square error of approximation
NFI: Normed fit index
CFI: Comparative fit index
TLI: Tucker-Lewis index
IFI: Incremental fit index
AVE: Average variance extracted values
CR: Combined reliability
KMO: Kaiser-Meyer-Olkin
