# Supplementary material for: Development and validation of a maternal anxiety for neonatal jaundice scale in China
Source: BMC Psychiatry. 2022 Aug 4;22:526. doi: 10.1186/s12888-022-04161-1 (PMC9351162; doi:10.1186/s12888-022-04161-1)
Supplement: Supplementary file 1 — Additional file 1. [file 12888_2022_4161_MOESM1_ESM.docx]

| **Table S1 Demographic characteristics of newborns (N=1127)** | |
| --- | --- |
| **Variables** | **n（%）/‾X±S** |
| **Newborn gender** |  |
| male | 565(50.13) |
| female | 562(49.87) |
| **Newborn age, day** | 3.32±2.53 |
| **gestational age, week** | 39.03±2.38 |
| Birth weight, g | 3204.88±446.59 |
| **Type of delivery** |  |
| Natural delivery | 722(64.06) |
| Cesarean section | 35.9(35.94) |

| **Table S2 The rotated factor loading matrix of 11-item MANJS** | | |
| --- | --- | --- |
| **Items** | **Factor loadings** | |
|  | **Factor 1** | **Factor 2** |
| c1 I am afraid that jaundice would threaten my child's health. | 0.29 | 0.68 |
| c2 I can hardly relax even though the doctor thinks the child's jaundice symptom is not serious. | **0.56** | **0.41** |
| c3 I loss sleep at night or have nightmares because I am worried about my child's jaundice. | 0.68 | 0.22 |
| c4 I go online for information about neonatal jaundice or keep consulting doctors and friends. | 0.15 | 0.86 |
| c5 I observe the child's every move and repeatedly confirm whether the behavior was related to jaundice. | 0.15 | 0.88 |
| c6 I keep an eye on the child's jaundice level and double check that it was within the normal range. | 0.18 | 0.83 |
| c7 I feel easily irritated until my child's jaundice subsides. | 0.83 | 0.24 |
| c8 I feel nervous until my child's jaundice subsides. | 0.87 | 0.25 |
| c9 I feel restless until my child's jaundice subsides. | 0.91 | 0.17 |
| c10 I have no appetite until my child's jaundice subsides. | 0.91 | 0.13 |
| c11 I can't concentrate on things because I am worried about my jaundiced child. | 0.88 | 0.13 |
| Eigenvalue | 5.92 | 1.96 |
| Variance explained (%) | 53.79 | 17.77 |
| Cumulative percentage (%) | 53.79 | 71.56 |

**Table S3 Inter-item correlation matrix**

|  | **c4** | **c5** | **c6** | **c7** | **c8** | **c9** | **c10** |
| --- | --- | --- | --- | --- | --- | --- | --- |
| **c4** | 1 | 0.74^**^ | 0.63^**^ | 0.32^**^ | 0.32^**^ | 0.28^**^ | 0.27^**^ |
| **c5** | 0.74^**^ | 1 | 0.75^**^ | 0.32^**^ | 0.35^**^ | 0.31^**^ | 0.28^**^ |
| **c6** | 0.63^**^ | 0.75^**^ | 1 | 0.38^**^ | 0.40^**^ | 0.33^**^ | 0.29^**^ |
| **c7** | 0.32^**^ | 0.32^**^ | 0.38^**^ | 1 | 0.83^**^ | 0.78^**^ | 0.70^**^ |
| **c8** | 0.32^**^ | 0.35^**^ | 0.40^**^ | 0.83^**^ | 1 | 0.86^**^ | 0.76^**^ |
| **c9** | 0.28^**^ | 0.31^**^ | 0.33^**^ | 0.78^**^ | 0.86^**^ | 1 | 0.84^**^ |
| **c10** | 0.27^**^ | 0.28^**^ | 0.29^**^ | 0.70^**^ | 0.76^**^ | 0.84^**^ | 1 |

Note: ^**^ *P*<0.01 (2-tailed).

| **Table S4 7-item MANJS CFA model fit indices in different random samples** | | | | | | | | | | | |
| --- | --- | --- | --- | --- | --- | --- | --- | --- | --- | --- | --- |
| **Samples** | **χ^2^** | **df** | **χ^2^/df** | **SRMR** | **RMSEA** | **GFI** | **AGFI** | **TLI** | **IFI** | **NFI** | **CFI** |
| N=500 | 45.071 | 13 | 3.467 | 0.033 | 0.070 | 0.998 | 0.995 | 0.935 | 0.960 | 0.945 | 0.960 |
| N=300 | 37.898 | 13 | 2.915 | 0.037 | 0.080 | 0.997 | 0.994 | 0.919 | 0.951 | 0.927 | 0.950 |
